# Nature-Based Interventions for Mental Health Care: Social Network Analysis as a Tool to Map Social Farms and their Response to Social Inclusion and Community Engagement

**DOI:** 10.3390/ijerph16183501

**Published:** 2019-09-19

**Authors:** Marta Borgi, Mario Marcolin, Paolo Tomasin, Cinzia Correale, Aldina Venerosi, Alberto Grizzo, Roberto Orlich, Francesca Cirulli

**Affiliations:** 1 Center for Behavioral Sciences and Mental Health, Istituto Superiore di Sanità, 00161 Rome, Italy; cinziacorreale@gmail.com (C.C.); aldina.venerosi@iss.it (A.V.); francesca.cirulli@iss.it (F.C.); 2 E-labora, 33170 Pordenone, Italy; mariomcln26@gmail.com (M.M.); paolo.tomasin@email.it (P.T.); 3 Healthcare Authority n. 5 “Friuli Occidentale”, 33170 Pordenone, Italy; alberto.grizzo@gmail.com (A.G.); roberto.orlich@aas5.sanita.fvg.it (R.O.)

**Keywords:** social farming, rural areas, mental health, social inclusion, job placement, social network analysis

## Abstract

Social farming represents a hybrid governance model in which public bodies, local communities, and economic actors act together to promote health and social inclusion in rural areas. Although relational variables are crucial to foster social farm performance, the relational system in which farms are embedded has still not been fully described. Using social network analysis, here we map the nature of the links of a selected sample of social farms operating in Northern Italy. We also explore possible network variations following specific actions taken to potentiate local social farming initiatives. The results show a certain degree of variability in terms of the extension and features of the examined networks. Overall, the actions taken appear to be significant to enlarge and diversify farms’ networks. Social farming has the potential to provide important benefits to society and the environment and to contrast vulnerability in rural areas. Being able to create social and economic networks of local communities, social farming may also represent an innovative way to respond to the cultural shift from institutional psychiatry to community-based mental health care. This study emphasizes the critical role played by network facilitation in diversifying actors, promoting heterogeneous relationships, and, in turn, system complexity.

## 1. Introduction

Environmental factors can play a fundamental role in mental health prevention and promotion [1,2,3,4]. Although research in this area has typically targeted environmental risk factors, more recent studies focus on the role of positive environmental conditions that may promote resilience and adaptation to stress [1,5,6]. In particular, exposure to natural landscapes or their composite features, such as plants and animals, is increasingly recognized as a source of relaxation and regeneration for humans [7]. One of the possible connections between access to natural environments and health has to do with increasing social relationships [8,9,10]. By providing an opportunity for social engagement, natural environments—including urban green spaces—may help to promote social integration, social ties, and a sense of community, reducing loneliness [11,12,13]. All of these factors are known to play a beneficial role in the maintenance of physical and mental health [5,14,15,16].

Considering the evidence accumulated so far, rural areas appear to be an elective place to promote health and to build social interventions for people with (or at risk for) mental disorders. Recently, the European Commission has highlighted the key role of rural areas—which account for a large part of the European territory and of the population of the Member States—in addressing the current and future societal challenges in terms of provision of public goods, environmental sustainability, and improved social wellbeing for both rural and urban inhabitants [17]. However, while being of great importance, rural areas are highly vulnerable; rural exodus and youth drain, geographical isolation, low educational attainments, scarcity of public resources, workforce shortages, and lack of appropriate models of health care, all represent considerable challenges to deliver appropriate health and social services for rural residents and to foster entrepreneurship in traditional rural domains [18,19,20,21,22,23]. The creation of hybrid governance models in which public bodies, local communities, and economic actors work together to co-produce social services may offer an innovative solution to buffer the financial (and organizational) challenges faced by the national health systems and to increase economic sustainability [24,25].

*Social farming* (SF) or *care farming*—is the term used to describe short or long-term farming activities to promote social inclusion of people with different disabilities and vulnerable target groups [26,27,28]. SF is playing a growing role in creating an independent local network of social support that, as a consequence, may sustain health-care institutions through practices involving vulnerable/disadvantaged people and embedded in local social contexts [28,29,30]. Using agricultural resources, such as animals and plants, SF is able to address specific social needs, including rehabilitation, sheltered employment, life-long education, and other activities that contribute to social inclusion [27,31]. Targets of SF initiatives are often people with physical or mental disabilities, long-term unemployment, or, more in general, people experiencing social exclusion. By being included in agricultural activities, such as horticulture, food processing, selling of products, animal care, and management of the farm-restaurant, they have the opportunity to increase their social and professional skills and to be integrated into society and the labor market. 

Together with its beneficial impact on the social, physical, and mental wellbeing of people, SF promotes and generates social services to local communities [30,32], strengthens the economic and social viability of rural communities [27], and fosters the farming sector and society in general [33,34]. Moreover, this practice allows farms to broaden and diversify their scope of activities [35], to provide new sources of income for the farming household, helping farmers to become more integrated into local communities [31,33,34,35].

Importantly, since SF is based on partnerships among public bodies, economic actors and local communities, the relational system in which farms are embedded is crucial to foster farm performance [36,37]. The network of alliances built with local actors is able to promote entrepreneurial dynamism and represent an advantage from the point of view of strategic autonomy and sustainability [38]. Recently, Bassi and colleagues [39] explored how structural (e.g., farm size) and relational variables (i.e., social, economic, and other relationships) affect the performance of farms engaged in SF in the Friuli Venezia Giulia Region of Italy. Their findings indicate that, more than structural variables, relational variables are directly and positively related to the ability of the farms to cope with market problems and to implement other activities, including the engagement of disadvantaged people. 

Although these data point to the importance of relational variables as a key point in SF success, the nature of the relational system in which social farms are embedded has still not been fully described, and there is very little knowledge on the appropriate measures needed to strengthen the relations and networks at the local level. This paper aims to address this gap in knowledge. The study presented here was conducted in the province of Pordenone (Friuli Venezia Giulia, Italy) and had two main objectives: (i) mapping and describing the nature of the networks of a selected sample of farms engaged in social inclusion activities involving people with mental health issues; (ii) exploring network variations as a result of specific actions taken to facilitate farms’ networking and to potentiate/foster local SF initiatives.

## 2. Materials and Methods 

### 2.1. Research Objectives and Procedure

This study had two main objectives. The first aim was to map and describe the nature of the networks of a selected sample of farms operating in the province of Pordenone (Friuli Venezia Giulia, Italy) involved in SF. We also sought to explore possible network variations following specific actions taken to facilitate farms’ networking and to potentiate/foster local SF initiatives (see Section 2.1.1). To these aims, we first (T0) analyzed the nature of the networks among farms and between farms and both public and private actors outside the SF environment using *Social Network Analysis* (SNA). Then specific actions were taken, as described below. After 10 months (T1), we analyzed again whether and how networks of the participating farms had changed as a result of the project, by collecting quantitative network variations. 

#### 2.1.1. Actions

A public-private collaboration between the Health care Authority AAS5 “Friuli Occidentale” (who sponsored the project), the Italian National Institute of Health (“Istituto Superiore di Sanità”, the leading technical and scientific public body of the Italian National Health Service), and the local Consortium of Social Cooperatives “Leonardo” was established. In the context of a collaborative project, different actions were taken: namely: (a) inclusion and engagement of a convenient sample of patients with mental health issues in selected local farms; (b) facilitation of the participating farms’ networking to promote and strengthen farms’ ties with the local community through the engagement of both private and public institutions (health, civil authorities, socio-economic actors); and (c) dissemination of local SF experiences and initiatives. 

Patients with mental health issues were selected by the Health care Authority ASS5 and were engaged as workers in the participating farm’s rural activities (e.g., horticulture, gardening, and domestic animals care), with the aim of supporting their employment and social inclusion in the SF context. The Health care Authority ASS5 was responsible for patients’ recruitment and monitored their engagement in farming activities. A territorial facilitator was in charge of connecting social/health services with the farms. 

The project partners acted actively to facilitate farms’ networking and to sensitize local communities to SF experiences and initiatives. Social events, including farmer’s markets, were organized in order to promote and strengthen farms’ ties with the local community through the engagement of health, municipalities, and socio-economic actors, volunteering associations, as well as citizens. 

### 2.2. Participants

#### 2.2.1. Farms’ Selection

Six farms were selected among the thirty already involved in SF in the province of Pordenone. A list of farms was obtained by the Forum of Social Farming of the province of Pordenone and by the ERSA, the Regional Agency for Rural Development, a public body founded by the autonomous region of Friuli Venezia Giulia. The study’s objectives required the collection of sociometric data. Due to the health care authority project constraints, convenience sampling was used. Farms were selected on the basis of the following criteria: (i) located in the province of Pordenone; (ii) availability to engage persons with mental health issues in farming activities; (iii) business-oriented activities (e.g., production and direct sale or marketing of products and services).

#### 2.2.2. Farm Description

Participating farms were located in the south-east of the Friuli Venezia Giulia region. Characteristics of the participating farms are described in Table 1.

### 2.3. Data Collection and Analysis

Data were collected at two different time points. We started by considering the networks already in place between the selected farms and other institutions before the launch of the project in 2010 (T0). Following this analysis, we investigated changes in the farms’ networks over the following 10 months (T1), mapping specifically those links allowing the participating farms to promote and facilitate social inclusion and job placement of persons with mental issues. 

Interview surveys of the owner or manager of each farm (not self-reporting) allowed collecting sociometric data for the quantitative analysis of the farms’ networks. Interviews consisted of a series of closed-ended questions designed to collect information on relational variables of the social farms, namely economic relations (customer-supplier ties), social relations (collaboration in social activities), and other types of relations/collaborations. Participants were asked about the number, location (i.e., in the same province, or in another province or in another region), legal status (i.e., public or private or non-profit sector), and sector of activity of the actors they have links with, as well as the number, nature, and frequency of these links. Links were also characterized for their economic (i.e., exchanges concerning or affecting material resource) vs. non-economic nature and for their impact on the persons with mental health issues engaged in farming activities. In particular, a link was considered to have a direct impact when at least one person with mental issues was present (as, for example, during social and recreational activities) or when a person with mental issues was the direct beneficiary of the action (as in the case of initiatives promoting job placement). 

Ten months later, participants were interviewed again to identify both the prior links and previously unmentioned links they had established. In the case of numeric values, differences between T0 and T1 have been computed as % change (i.e., percent of the value at T0); in the case of percentage values, changes were computed as % points (i.e., the arithmetic difference of two percentages). 

The interviews took place on-site, lasted on average 60 min, and were set up by prior contact via email and telephone so that respondents were aware of the objectives of the study and the type of information to be collected. 

#### Social Network Analysis

*Social Network Analysis* (SNA) [40,41,42,43] was used to depict and describe the social networks of each participant at the two time points. SNA is based on mapping and characterizing the relationships that are established as a result of the interactions between different actors and groups of actors. In the SNA, the *nodes* in the network are the actors and/or groups of actors, while the *links* show relationships through flows of goods, information, and implementation of joint activities or other bonds between the nodes. In this study, relationships among farms and between farms and both public and private groups/organizations were described. We limited our analysis to the so-called *ego network*, namely the individual network of each participating farm. An ego network consists of the focal node (*ego*, i.e., the farm) and the nodes to whom ego is directly connected to (called *alters*), plus the *links* among the nodes. We thus limited our analysis to the description of the links (i.e., relationships or flows) between the ego and the alters, while we did not record *links* among alters. The relations among the surveyed farms were investigated too: data on the absence/presence of each farm’s *links* with other social farms were collected, as well as sharing of alters. The software Ucinet-NetDraw [44] was used for visualizing the farms’ networks.

## 3. Results

The Results of farms’ social networks are presented in three subsections. The first subsection characterizes the farms’ ego network and its members at T0. In the second subsection, we analyze changes of the networks over time, in particular by comparing two different time points (T0−T1). In the third section, relations among the surveyed farms and alter sharing is reported.

### 3.1. Description of the Ego Networks

#### 3.1.1. Nodes

Table 2 shows the number of nodes (alters) that the surveyed farms (egos) referred to be linked to, split by location and legal status. At the time T0, the number of nodes varied from 22 to 75 in the different participating farms. Most of the nodes were located in the Pordenone province (from 58.6% to 91.1% of the total nodes reported). The other nodes were located in another province (from 1.8% to 18.9%) or region (from 6.1% to 38.1%). Farms made links with actors in the public sector (from 0.0% to 41.3% of the total nodes reported), in private (from 33.9% to 90.0%), and in the private non-profit sector (from 10.0% to 47.9%). 

In Table 3, alters are identified according to their main sector of activity. On average, the most represented sector of activity is the tertiary or service sector, including health and social services (from 0.0% to 50.0% of the farms’ contacts), while nodes in the agricultural production represent less than one-third (from 2.4 % to 20.4%) of the farms’ reported contacts. Only a small proportion of alters was active in the social farming sector (from 0.0 to 8.0%) or in the education sector (except for farm D).

#### 3.1.2. Links

As shown in Table 4, at the time T0 the number of links the ego made with alters varied from 25 to 88 in the different interviewed farms, with an average link per node ranging between 1.14 and 1.47. Except for two farms (B 37.9% and F 45.8%), economic (for profit) links—i.e., exchanges concerning or affecting material resources, such as buying and selling goods and services—represented more than half (varying from 55.7% to 91.7%) of the reported links. Only a small proportion of the links made by participants (range: 3.5%−25.8% in the different farms) represented “high-frequency contacts,” namely daily and weekly contacts (vs. monthly, seasonal, or occasional contacts). Except for two farms (C and D), participants reported a high proportion of links with a “direct” impact on the persons with mental health issues engaged in farming activities (from 56.9% to 80.0%) (Table 4). 

Most of the links made by the participants with alters were represented by ties aimed at buying and selling goods and services (from 37.5% to 91.7%). Other links were represented by flows of information (from 0.0% to 9.7%) and non-economic resources and services (from 0.0% to 18.1%), educational activities (from 0.0% to 36.4%), initiatives promoting autonomy, social inclusion, and job placement of persons with mental issues (from 0.0% to 20.0%), joint social activities (e.g., public social events; from 0.0% to 10.6%) and research and evaluation (clinical studies, rehabilitation interventions, and research; from 0.0% to 4.2%) (details are shown in Table 5).

### 3.2. Changes in the Farms’ Networks

Changes of the networks over time were analyzed by comparing two different time points (T0−T1). Changes are shown as % increase/decrease (in the case of numeric values) and as % point variation (in the case of percentage values). The results presented are relative to five of the six participating farms, since one of the farms (i.e., farm E) did not participate in the data collection at T1. 

#### 3.2.1. Nodes

As shown in Table 6, in all the study sites the number of nodes (alters) increased over the study period (T0–T1), with a percent increase varying from 1.3% to 89.8% in the different participating farms. The percentage of local actors (i.e., nodes located in the province of Pordenone), which represented the majority of the reported nodes at T0 slightly increased (from 0.6 to 4.4% points) over the study period, except for one farm (farm A) in which the proportion of alters in the same province decreased, while new contacts were made with actors in another province and in another region. The percentage of nodes in the public, private, and private nonprofit sectors changed over time, with variations going from −12.0 to +8.2 percentage points. In particular, the proportion of nodes in the public sector increased in all farms’ networks, while the presence of actors in the private sector slightly increased in only two farms (farms B and F). The proportion of nodes in the nonprofit sector increased only in one participant (farm C, a sole proprietorship), with a variation of 8.2 percentage points.

After 10 months, all participating farms reported a slight increase in the percentage of network members in the education sector (range: 1.8–6.9). Three farms reported a slight increase in the percentage of nodes in the health and social services sector (range: 2.1–4.1); of these, two were sole proprietorships. Two of the participating farms (A, a social cooperative and C, a sole proprietorship) reported a slight decrease (−2.5 and −1.3 percentage points, respectively) of nodes in the agricultural production sector. Other variations are shown in Table 7.

#### 3.2.2. Links

As shown in Table 8, the number of network links reported by all surveyed farms increased over the study period (T0−T1) (% increase range: 5.6–104.2). In different farms, this was combined either with an increase in the average links per node (when the number of links increased proportionally more than the number of nodes) or with a decrease in the average links per node (when the number of nodes increased proportionally more than the number of links). The proportion of the economic links (on the total reported) decreased over the study period in all participating farms (variation range: −1.5−−7.4% points). The percentage of high frequency links slightly changed over time, with variations going from −6.6 to +9.4 percentage points. Except for two farms (B and F), participants reported a higher proportion of links with a “direct” impact on the persons with mental health issues engaged in farming activities (increase range: 4.9–6.6% point variation).

Percentage of links aimed at buying and selling goods and services—which represented the major proportion at time T0—as well as flows of non-economic resources and services decreased in all participating farms. Except for one farm (farm F), the proportion of links related to educational activities also decreased. By contrast, the actions taken had the effect of increasing the networks’ links related to initiatives promoting social inclusion and job placement (with the only exception of farm A), research and evaluation (except for farm A), and flows of information (except for farm D). Contrary to what was expected, three of the five participating farms reported a decrease in the proportion of the links related to joint social activities. More details are shown in Table 9.

### 3.3. Relations among Social Farms and Sharing of Alters

**Figure 1:**
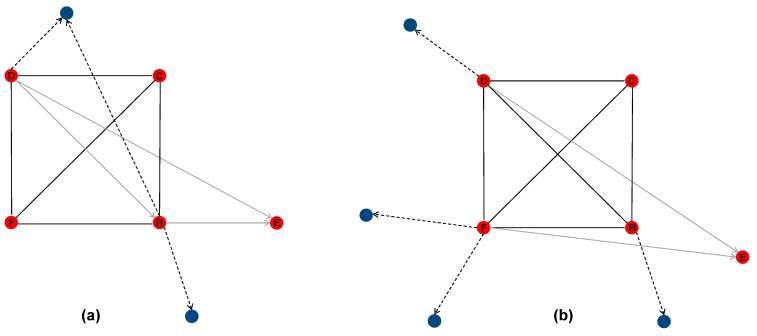
The network of contacts between the interviewed farms and between them and other social farms in the area at T0 (**a**) and T1 (**b**). Circles represent the nodes. The red circles are the egos (the interviewed farms), and the blue circles represent other social farms that were referred to be linked to. Lines represent the links connecting pairs of nodes. The black lines represent links indicated by both actors; the grey arrows represent links indicated only by one of the two actors. When only one of the two actors was interviewed (ties with other social farms in the area), links are represented by dash lines.

First, we explored connections among the participating farms (egos) and between them and other social farms in the area. Except for two, all the interviewed farms reported to have links with some of the other egos (range: 2–4), as well as with other (n = 2) social farms in the area (Figure 1a). Changes observed over the study period are shown in Figure 1b.

**Figure 2:**
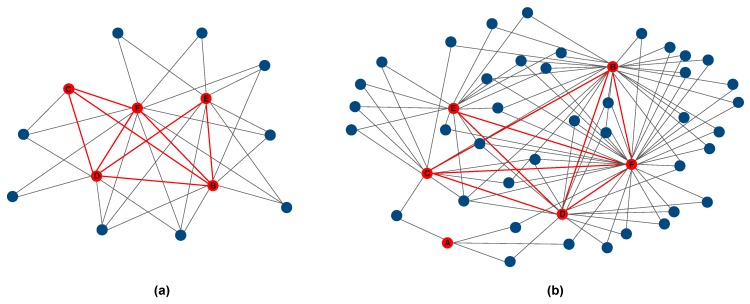
Sharing of alters among the interviewed farms at T0 (**a**) and T1 (**b**). Circles represent the nodes. The red circles are the egos (the interviewed farms), and the blue circles represent the alters. Lines represent the links connecting the pairs of nodes. The red lines represent links between the interviewed farms.

Second, we analyzed whether the ego networks of the participating farms were connected through sharing of alters. We considered two farms as directly sharing alters when they identify the same node in their ego networks. At T0 (Figure 2a) the survey identified nine shared alters, which represent less than 3% of the total nodes reported by the participating farms. At T1 (Figure 2b), shared alters were 40 (representing 10% of the total at T1).

## 4. Discussion

The aim of this study was to describe the social and economic relationships of a sample of farms involved in SF and to explore whether the engagement of persons with mental health issues in their activities, combined with dissemination and promotion actions, is able to affect their network. This information is of particular relevance, considering that relational variables are crucial to enhance social farms’ performance and, thus, for the functioning of the system itself [36,37,39].

Although the number of social farms included in the survey was small, we still observed a certain degree of variability in terms of activities proposed (both agricultural and social), status (four social cooperatives and two sole proprietorships) and in terms of the extension and features of their networks. 

Overall, at T0, the network of relations in which the surveyed farms were embedded did not appear to be particularly developed. Participants reported a number of nodes varying from 22 to 75, mostly based in the same province (local networks) and represented by private actors. The proportion of public institutions in the farms’ networks was less than one-third of the total nodes reported, except in a case study (farm D, a sole proprietorship), whose network at T0 was the largest (in terms of the number of nodes) and included a similar proportion of public and private institutions. As for the contacts with institutions operating in the non-profit sector, these were particularly scarce in the case of the two sole proprietorships (farms C and D), for which nodes in the third sector represented about 10% of the total. Consistently with the farms’ involvement with actors in the private sector—mostly tertiary/service, trade, and agricultural sectors—at T0 reported links of the interviewed farms were mostly represented by economic exchanges, i.e., buying and selling goods and services. In four of the six participating farms, the economic links accounted for more than half of the total, while in farms B and F, two social cooperatives, economic links represented respectively 38% and 46% of the total reported links. 

Hence, the overall picture at T0 indicates an entrepreneurial/business vocation (e.g., production and direct sale or marketing of products and services) of the selected farms and diverse income flows deriving from the various multifunctional practices. This could be crucial, especially for small farmers, providing the income required to enable them to stay in business and reduce the risk of dependence on public funding. Through the building of new socio-economic relationships, SF is able to create market opportunities, thus representing an important source of diversification for farmers and a potential new source of income for the farming household [35,45,46].

As expected, the aim of rural production appears to be well conjugated with the pursuit of social ends in the case of social cooperatives, which reported a relatively high proportion of health and social services in their network (range: 12–50% in the different farms). By contrast, the two sole proprietorships (farms C and D) reported a very low proportion of nodes active in this sector, though farm D appears particularly active in the education sector. Except for this latter case, all interviewed farms reported a very low proportion of nodes active in the education sector, as well as a low proportion of links represented by educational activities. This can be viewed as a limit, considering that promoting (or generating) education services represents the first step towards the inclusion of people with ‘low contractual capacity’ as those with mental and physical disabilities, migrants, and other people experiencing social exclusion [27]. Indeed, SF programs have the potential to represent a driver for the provision of suitable local training, education, and capacity-building for people in rural localities to undertake local initiatives, ultimately contrasting low educational attainment and youth exodus characterizing rural areas [45]. Through the contribution of professionals in various fields (psychiatrists, legal consultants, marketing experts, researchers), education activities are also crucial to create a strong foundation for the SF sector, to be built through guidelines, examples of best practices and quality criteria [27,31,45].

Interactions among participating farms and between them and other social farms in the area, as reported at T0, appear extremely weak. Only some of the interviewed farms (four of the six participating) reported to have links with other social farms in the area, and the number of social farms in their network was very low (range: 2–6). Consolidated links between social farms–particularly in the form of collaboration in rehabilitation and job placement activities for disadvantaged people–could contrast entrepreneurial vulnerability. By creating networking opportunities and providing access to new resources, relations with other farms might, in fact, support the smallest (and more vulnerable) social farms and help them to improve their performance [27,39]. 

Overall, actions taken in the context of the present study–including the engagement of both private and public institutions (health, civil authorities, socio-economic actors), as well as dissemination strategies, appear to be significant to enlarge and diversify social farms’ network. In all the participating farms, both the number of nodes and the number of links increased over the study period. The increase was more evident for the social cooperatives than for the two sole proprietorships (C and D). Moreover, the specific actions taken resulted in changes both in the networks’ structure and in the flow within the networks. Overall, changes appear to be in the direction of a greater balance between economic and social activities. As an example, in comparison with T0, at T1 all examined ego networks were characterized by a higher proportion of non-economic exchanges, as well as of actors active in the educational sector (both non-economic links and nodes in the education sector were scarce at T0). Farms C and D (two sole proprietorships) reported the highest (although still moderate) increase of health and social services in their network, while the presence of contacts with health and social services decreased in the network of farms already reporting a high presence of nodes in this sector at T0, e.g., farm B. Consistently, at T1 farm B reported a higher proportion of nodes in the agricultural production in comparison with T0. Indirect empowerment, due to the participation in the project, might have contributed to the general growth of the farms, which is, in any case, a positive result of the activities proposed. As expected, actions taken actively encouraged initiatives promoting social inclusion and job placement, as well as initiatives linked to research and evaluation. Furthermore, the proportion of links with a direct impact on persons with mental health issues increased over the study period, particularly in the two farms (C and D) reporting the lowest percentage of these links at T0. Finally, it is important to emphasize that the intervention changed the size of shared networks among the farms, creating a greater number of shared contacts and much more complex territorial inter-relationships.

In general, our results emphasize the critical role played by network facilitation in diversifying actors, promoting heterogeneous relationships, and, in turn, system complexity. Hence, agricultural innovation policies should foster the emergence and functioning of connections among different actors involved in SF in order to build appropriate linkages and facilitate multi-stakeholder interactions [47,48].

The research has some limitations. The description of a few cases limits representativeness. Future research should widen the sample size and look at SF experiences in different areas and countries across and outside the European continent. Although the use of SNA for mapping, measuring and analyzing social relationships between people, groups and organizations appear a suitable method to investigate the nature—and variation—of the networks of farms engaged in social inclusion, the analysis of the ego networks has methodological limitations. The main limitation is related to the bias of reports. Analyses relied on the accuracy of reports from the focal actor, the ego, while we did not observe the status of relationships from the perspective of the alters. Moreover, since we focused only on links between the ego and the alters, we did not explore the links between the alters, although those links can potentially affect the ego. Lastly, we cannot exclude that other concomitant events (e.g., rural policies, funding) might have contributed to the changes observed. 

## 5. Conclusions and Further Directions

According to Organisation for Economic Co-operation and Development (OECD) (2018) [49], one in six people in the EU is affected by some sort of mental health problem; this has an estimated total cost of over EUR 600 billion, exceeding 4% of the EU Gross Domestic Product (GDP). Moreover, the increased flow of migration is putting additional pressure on the EU’s inclusion policies. Recent studies have shown that nature-based private-public partnerships such as social agriculture and urban green infrastructures are providing cost-effective solutions to the above-mentioned trends [28]. The agricultural sector has become particularly aware of the multifunctional character of the land and, although the core aim for agriculture remains the production of primary products such as food, fiber, and oil, it also provides other important benefits to society and the environment. In line with the recommendations of the World Health Organization’s (WHO) Mental Health Action Plan 2013–2020 [50], by providing de-institutionalized care, SF may represent an innovative way to respond to the cultural shift from institutional psychiatry to community-based mental health care. Moreover, the promotion and strengthening of bottom-up approaches able to create social and economic networks of local communities have been pointed out as an essential element to contrast vulnerability and fighting poverty in rural areas [51]. 

This paper contributes to our understanding of social farm’s networks by exploring how and to which extent they become embedded in the local network of actors. Since relational variables appear to represent the driving force affecting social farm performance [39], these results may help policymakers and practitioners to promote SF initiatives. 

Changes in socio-economic networks of social farms should, in the future, be analyzed in conjunction with their impact on persons included in SF initiatives. Considering the recent surge of interest in the potential of natural environments and nature-based interventions in contributing to the prevention and mitigation of mental disorders or states, SF may be viewed as an “open-air” laboratory to further explore evidence of an association between contact with nature and mental health and to identify the mechanisms underlying this link [52,53,54,55]. We are currently developing specific questionnaires for evaluating potential changes in the social network that the single individual may evolve after his/her involvement in SF programs and analyzing whether structural and relational variables of the farm have an impact on social inclusion and job placement of persons with mental issues. Measuring whether and to what extent SF initiatives help in reducing the burden on health and social care systems also appears crucial.

## Figures and Tables

**Table 1:** Characteristics of the participating farms.

Farm	Legal Status	Agricultural (and Social) Activities
A	Social cooperative	Crop farming and Horticulture, Animal husbandry, Farm education
B	Social cooperative	Horticulture, Animal husbandry, Farm education
C	Sole proprietorship	Crop farming, Floriculture and Horticulture, Animal husbandry and breeding
D	Sole proprietorship	Viticulture, Farmhouse, Animal husbandry, Rural tourism
E	Social cooperative	Crop farming, Direct sale/marketing of products
F	Social cooperative	Crop farming, Floriculture and Horticulture, Animal husbandry and breeding; Animal-Assisted Interventions

**Table 2:** Number, location and legal status of the nodes (time point: T0)

Farms	Total n. of Nodes (Alters)	Location (%)			Legal Status (%)		
		In the Same Province	In Another Province	In Another Region	Public Sector	Private Sector	Non-profit Sector
A	61	73.6	18.9	7.5	8.3	60.0	31.7
B	56	91.1	1.8	7.1	25.0	33.9	41.1
C	30	58.6	3.3	38.1	0.0	90.0	10.0
D	75	64.0	12.0	24.0	41.3	46.7	12.0
E	22	77.3	4.5	18.2	18.2	40.9	40.9
F	49	81.6	12.2	6.1	10.4	41.7	47.9

**Table 3:** The sector of activity of the nodes (time point: T0).

Farm	Sector of Activity (%)								
	Education	Agricultural Production	Industrial Production	Trade	Social Farming	Health and Social Services	Food Services and Hotels	Other Services	Other Sectors
A	2.0	20.4	2.0	28.6	0.0	12.2	2.0	0.0	32.7
B	0.0	2.4	0.0	11.9	7.1	50.0	4.8	21.4	2.4
C	na	20.0	0.0	30.0	6.7	0.0	23.3	20.0	0.0
D	40.0	0.0	0.0	21.3	8.0	4.0	0.0	26.7	0.0
E	4.5	18.2	0.0	22.7	0.0	27.3	0.0	27.3	0.0
F	0.0	10.2	6.1	2.0	6.1	18.4	0.0	51.0	6.1

na: not available (the proportion of nodes in the educational sector is not accurate since this participant did not report the exact number of schools engaged).

**Table 4:** The number of links, average links per node, and % of economic links, high-frequency links, and links with a direct impact on the persons with mental issues (time point: T0).

Farms	Total n. of Links	Average Linksper Node	Economic Links (%)	High-Frequency Links (%) *	Links with Direct Impact (%) ^#^
A	85	1.39	64.7	3.5	67.1
B	66	1.18	37.9	25.8	60.6
C	36	1.20	91.7	22.2	0.0
D	88	1.17	55.7	9.1	10.2
E	25	1.14	80.0	16.0	80.0
F	72	1.47	45.8	19.4	56.9

* daily and weekly contacts; ^#^ link with a direct impact: when at least one person with mental health issues was present or was the direct beneficiary of the action.

**Table 5:** Nature of the links made by participants with the reported alters (time point: T0).

Farm	Buying and Selling Goods and Services	Flows of Non-economic Resources and Services	Educational Activities	Initiatives Promoting Social Inclusion and Job Placement	Joint Social Activities	Flows of Information	Research & Evaluation	Other *
A	64.7	11.8	2.4	1.2	10.6	3.5	3.5	2.4
B	na	na	na	na	na	na	na	na
C	91.7	2.8	2.8	0.0	2.8	0.0	0.0	0.0
D	55.7	0.0	36.4	2.3	0.0	3.4	0.0	2.3
E	80.0	0.0	0.0	20.0	0.0	0.0	0.0	0.0
F	37.5	18.1	1.4	5.6	9.7	9.7	4.2	13.9

na: not available (this participant did not report the nature of the links made with alters); * tourism services (hospitality, transport, attractions), strategic actions (e.g., product and service promotion), donation/solidarity.

**Table 6:** Variation over time (T0−T1) in the number of nodes, their location, and legal status.

Farms	Total n. of Nodes (% Increase)	Location (% Points Variation)			Legal Status (% Points Variation)		
		In the Same Province	In Another Province	In Another Region	Public Sector	Private Sector	Non-profit Sector
A	27.9	−5.7	4.2	1.5	6.2	−4.8	−1.4
B	28.6	0.6	−0.4	−0.2	1.5	1.4	−2.9
C	6.7	2.7	−0.2	−0.6	3.0	−11.2	8.2
D	1.3	4.4	−2.8	−1.6	2.1	−1.9	−0.2
F	89.8	2.3	−6.8	4.5	7.0	5.0	−12.0

**Table 7:** Variation (expressed as percentage points) over time (T0−T1) in the sector of activity of the network nodes.

Farm	Sector of Activity (% Point Variation)								
	Education	Agricultural Production	Industrial Production	Trade	Social Farming	Health and Social Services	Food Services and Hotels	Other Services	Other Sectors
A	1.8	−2.5	−0.8	−4.2	0.0	−3.3	1.8	39.7	−32.7
B	6.9	4.6	0.0	−5.0	−1.6	−15.3	−2.0	14.7	−2.4
C	na	−1.3	0.0	16.9	2.7	3.1	−1.5	−20.0	0.0
D	1.9	1.4	0.0	−5.1	−1.2	4.1	0.0	−1.0	0.0
F	4.3	1.6	−4.0	5.5	0.3	2.1	0.0	−6.9	−2.9

na: not available (the proportion of nodes in the educational sector is not accurate since this participant did not report the exact number of schools engaged).

**Table 8:** Variation over time (T0−T1) in the number of links, average links per node, and the % of economic links, high-frequency links, and links with direct impact on the persons with mental issues.

Farms	Total n. of Links (% Increase)	Average LinksPer Node (% Increase)	Economic Links (% Point Variation)	High-frequency Links (% Point Variation) *	Links with Direct Impact (% Point Variation) ^#^
A	34.1	5.1	−1.5	5.3	6.6
B	24.2	−3.5	−7.4	−6.6	−3.1
C	5.6	−1.0	−4.9	9.4	5.3
D	5.7	4.6	−4.1	−0.5	4.9
F	104.2	7.5	−6.3	−3.6	−11.0

* daily and weekly contacts; # link with a direct impact: when at least one person with mental health issues was present or was the direct beneficiary of the action.

**Table 9:** Variation (expressed as percentage points) over time (T0−T1) in the nature of the links made by the participants with the reported alters.

Farm	Buying and Selling Goods and Services	Flows of Non-economic Resources and Services	Educational Activities	Initiatives Promoting Social Inclusion and Job Placement	Joint Social Activities	Flows of Information	Research & Evaluation	Other *
A	−1.5	−11.8	−0.6	−1.2	−9.7	23.7	−2.7	3.8
B	na	na	na	na	na	na	na	na
C	−4.8	−2.8	−0.1	0.0	−2.8	2.6	2.6	5.3
D	−4.1	0.0	−2.0	3.1	4.3	−3.4	0.0	2.0
F	−3.5	−12.6	2.0	6.7	−0.9	3.2	6.0	−1.0

na: not available (this participant did not report the nature of the links made with alters); * tourism services (hospitality, transport, attractions), strategic actions (e.g., product and service promotion), donation.

## References

[1] Meyer-Lindenberg A., Tost H. (2012). Neural mechanisms of social risk for psychiatric disorders. Nat. Neurosci..

[2] Peen J., Schoevers R.A., Beekman A.T., Dekker J. (2010). The current status of urban-rural differences in psychiatric disorders. Acta Psychiatr. Scand..

[3] Kovess-Masféty V., Alonso J., de Graaf R., Demyttenaere K. (2005). A European Approach to Rural—Urban Differences in Mental Health: The ESEMeD 2000 Comparative Study. Can. J. Psychiat..

[4] Christmas J.J. (1973). Psychological stresses of urban living: New direction for mental health services in the inner city. J. Natl. Med. Assoc..

[5] Tost H., Champagne F.A., Meyer-Lindenberg A. (2015). Environmental influence in the brain, human welfare and mental health. Nat. Neurosci..

[6] Russo S.J., Murrough J.W., Han M.H., Charney D.S., Nestler E.J. (2012). Neurobiology of resilience. Nat. Neurosci..

[7] Mantler A., Logan A.C. (2015). Natural environments and mental health. Adv. Integr. Med..

[8] Kuo M. (2015). How might contact with nature promote human health? Promising mechanisms and a possible central pathway. Front. Psychol..

[9] Hartig T., Mitchell R., De Vries S., Frumkin H. (2014). Nature and health. Annu. Rev. Public Health.

[10] Maas J., Van Dillen S.M., Verheij R.A., Groenewegen P.P. (2009). Social contacts as a possible mechanism behind the relation between green space and health. Health Place.

[11] Kaźmierczak A. (2013). The contribution of local parks to neighbourhood social ties. Landscape Urban Plan..

[12] De Vries S., Van Dillen S.M., Groenewegen P.P., Spreeuwenberg P. (2013). Streetscape greenery and health: Stress, social cohesion and physical activity as mediators. Soc. Sci. Med..

[13] Sugiyama T., Leslie E., Giles-Corti B., Owen N. (2008). Associations of neighbourhood greenness with physical and mental health: Do walking, social coherence and local social interaction explain the relationships?. J. Epidemiol. Commun. Health.

[14] Holt-Lunstad J., Smith T.B., Layton J.B. (2010). Social relationships and mortality risk: A meta-analytic review. PLoS Med..

[15] Nieminen T., Martelin T., Koskinen S., Aro H., Alanen E., Hyyppä M.T. (2010). Social capital as a determinant of self-rated health and psychological well-being. Int. J. Public Health.

[16] House J.S., Landis K.R., Umberson D. (1988). Social relationships and health. Science.

[17] European Union European Conference on Rural Development, Cork 2.0 Declaration 2016 A Better Life in Rural Areas. https://enrd.ec.europa.eu/sites/enrd/files/cork-declaration_en.pdf.

[18] Bourke L., Humphreys J.S., Wakerman J., Taylor J. (2012). Understanding rural and remote health: A framework for analysis in Australia. Health Place.

[19] Sibley L.M., Weiner J.P. (2011). An evaluation of access to health care services along the rural-urban continuum in Canada. BMC Health Serv. Res..

[20] Belanger K., Stone W. (2008). The social service divide: Service availability and accessibility in rural versus urban counties and impact on child welfare outcomes. Child Welfare.

[21] Smith K.B., Humphreys J.S., Wilson M.G. (2008). Addressing the health disadvantage of rural populations: How does epidemiological evidence inform rural health policies and research?. Aust. J. Rural Health.

[22] Serneels P.M., Lindelow M., Garcia-Montalvo J., Barr A., Abigail A. Health Policy Planning Forthcoming. UPF Working Paper Series 2006. https://ssrn.com/abstract=1002563.

[23] Dixon J., Welch N. (2000). Researching the rural–metropolitan health differential using the ‘social determinants of health’. Aust. J. Rural Health.

[24] García-Llorente M., Rossignoli C., Di Iacovo F., Moruzzo R. (2016). Social farming in the promotion of social-ecological sustainability in rural and periurban areas. Sustainability.

[25] Di Iacovo F., Moruzzo R., Rossignoli C. Social farming and social innovation in the perspective of new rural policies. Proceedings of the 2nd International Conference on Agriculture in an Urbanizing Societies.

[26] Haubenhofer D.K., Elings M., Hassink J., Hine R.E. (2010). The development of green care in western European countries. Explore.

[27] Di Iacovo F., O’Connor D. (2009). Supporting policies for social farming in Europe. Progressing Multifunctionality in Responsive Rural Areas. https://arpi.unipi.it/handle/11568/132812#.XYLzDmYRXIU.

[28] Sempik J., Hine R., Wilcox D. (2010). Green Care: A Conceptual Framework, A Report of the Working Group on the Health Benefits of Green Care.

[29] Hine R. (2008). Care farming: Bringing together agriculture and health. Ecos.

[30] Hine R., Peacock J., Pretty J. (2008). Care farming in the UK: Contexts, benefits and links with therapeutic communities. Therapeutic Commun..

[31] Hassink J., Van Dijk M. (2006). Farming for Health: Green-care farming across Europe and the United States of America.

[32] Leck C., Evans N., Upton D. (2014). Agriculture–Who cares? An investigation of ‘care farming’in the UK. J. Rural Stud..

[33] Pascale A. (2010). Linee Guida per Progettare Iniziative di Agricoltura Sociale.

[34] Senni S. (2007). Competitività dell’impresa agricola e legame con il territorio: il caso dell’agricoltura sociale. Agriregionieuropa.

[35] Van der Ploeg J.D., Roep D. (2003). Multifunctionality and rural development: the actual situation in Europe. Multifunct. Agric. New Paradigm Eur. Agric. Rural Dev..

[36] Leeuwis C. (2013). Communication for Rural Innovation: Rethinking Agricultural Extension.

[37] Knickel K., Brunori G., Rand S., Proost J. (2009). Towards a better conceptual framework for innovation processes in agriculture and rural development: From linear models to systemic approaches. J. Agr. Educ. Ext..

[38] Fazzi L. (2011). Social co-operatives and social farming in Italy. Sociol. Ruralis.

[39] Bassi I., Nassivera F., Piani L. (2016). Social farming: A proposal to explore the effects of structural and relational variables on social farm results. Agri. Food Econ..

[40] Prell C. (2012). Social Network Analysis: History, Theory and Methodology.

[41] Hanneman R.A., Riddle M. (2005). Introduction to Social Network Methods.

[42] Krebs V. An Introduction to Social Network Analysis. http://www.orgnet.com/sna.html.

[43] Wasserman S., Faust K. (1994). Social Network Analysis: Methods and Applications.

[44] Borgatti S.P., Everett M.G., Freeman L.C. (2002). Ucinet 6 for windows: Software for social network analysis. Harv. MA Analytic Technol..

[45] O’Connor D., Lai M., Watson S. Overview of Social Farming and Rural Development Policy in Selected EU Member States. http://enrd.ec.europa.eu/enrd-static/fms/pdf/.

[46] Bassi I., Nassivera F., Piani L. (2016). Market opportunities for social farms. Riv. di Economia Agraria.

[47] Klerkx L., Hall A., Leeuwis C. (2009). Strengthening agricultural innovation capacity: Are innovation brokers the answer?. Int. J. Agri. Resour. Governance Ecol..

[48] Klerkx L., Aarts N., Leeuwis C. (2010). Adaptive management in agricultural innovation systems: The interactions between innovation networks and their environment. Agric. Syst..

[49] OECD/EU (2018). Health at a Glance: Europe 2018: State of Health in the EU Cycle.

[50] World Health Organization Mental Health Action Plan 2013–2020. https://apps.who.int/iris/handle/10665/89966.

[51] Bertolini P., Montanari M., Peragine V. Poverty and Social Exclusion in Rural Areas. Final Study Report 2008. https://iris.unimore.it/retrieve/handle/11380/606205/209247/rural_poverty_en.pdf.

[52] Haluza D., Schönbauer R., Cervinka R. (2014). Green perspectives for public health: A narrative review on the physiological effects of experiencing outdoor nature. Int. J. Environ. Res. Public Health.

[53] Bratman G.N., Hamilton J.P., Daily G.C. (2012). The impacts of nature experience on human cognitive function and mental health. Ann. N. Y. Acad. Sci..

[54] Maas J., Verheij R.A., Groenewegen P.P., De Vries S., Spreeuwenberg P. (2006). Green space, urbanity, and health: How strong is the relation?. J. Epidemiol. Commun. Health.

[55] Frumkin H. (2001). Beyond toxicity: Human health and the natural environment. Am. J. Prev. Med..

